# 3D Measurement of Human Chest and Abdomen Surface Based on 3D Fourier Transform and Time Phase Unwrapping

**DOI:** 10.3390/s20041091

**Published:** 2020-02-17

**Authors:** Haibin Wu, Shuang Yu, Xiaoyang Yu

**Affiliations:** The Higher Educational Key Laboratory for Measuring & Control Technology and Instrumentations of Heilongjiang Province, Harbin University of Science and Technology, Harbin 150080, China; woo@hrbust.edu.cn (H.W.); yuxiaoyang@hrbust.edu.cn (X.Y.)

**Keywords:** 3D measurement, fringe projection, 3D Fourier transform, phase unwrapping, phase measurement

## Abstract

Monitoring respiratory movements is an effective way to improve radiotherapy treatments of thoracic and abdominal tumors, but the current approach is limited to measuring specific points in the chest and abdomen. In this paper, a dynamic three-dimensional (3D) measurement approach of the human chest and abdomen surface is proposed, which can infer tumor movement more accurately, so the radiotherapy damage to the human body can be reduced. Firstly, color stripe patterns in the RGB color model are projected, then after color correction, the collected stripe image sequences are separated into the three RGB primary color stripe image sequences. Secondly, a fringe projection approach is used to extract the folded phase combined 3D Fourier transform with 3D Gaussian filtering. By the relationship between adjacent fringe images in the time sequence, Gaussian filter parameters with individual characteristics are designed and optimized to improve the accuracy of wrapped phase extraction. In addition, based on the difference between the fractional parts of the folded phase error, one remainder equation can be determined, which is used for time-phase unwrapping. The simulation model and human experiments show that the proposed approach can obtain the 3D image sequences of the chest and abdomen surface in respiratory motion effectively and accurately with strong anti-interference ability.

## 1. Introduction

During radiotherapy, respiratory movements can cause tumors and normal tissues of the chest and abdomen to move at a certain frequency and amplitude. Sometimes, respiratory movements may affect the radiotherapy effect and even cause radiotherapy damage to the human body. In order to solve this respiratory motion problem, now the most effective real-time tracking method is to monitor extracorporeal respiratory movement. Based on extracorporeal respiratory movement, the respiratory movement of the tumor can be inferred, and then, the relative position of the target area and the field can be controlled by the radiotherapy system [1].

The fact that the respiratory movement of the tumor can be deduced from extracorporeal respiratory movement has been proven to be effective [2]. Based on this premise, dynamic 3D measurement of the chest and abdomen surface can be used to infer tumor movement more accurately [3].

Currently, optical measurement has become the most practical method to solve the problem of dynamic 3D measurement of the chest and abdomen surface. The optical method contains the following three types, point imaging, line imaging, and surface imaging, of which the surface imaging method is the best choice for dynamic 3D measurement. One image or many images can be captured by the surface imaging method. Single image acquisition methods include binocular vision [4], spatially encoded light [5], Fourier transform profile [6], etc. Multiple image acquisition methods include the phase-shift profile [7], modulation measurement profile [8], etc. Due to the fact that the multiple image acquisition method needs to capture many images, it has low efficiency and is not suitable for dynamic measurements. Thus, the one image acquisition method is a good solution for dynamic three-dimensional measurement.

Among these single image acquisition methods, the binocular vision method needs complex stereo matching and has low accuracy [9]; and the spatially encoded light method needs to be coded and decoded by the neighboring pixels, which makes the measurement resolution limited and may cause measurement failure in the case of surface height jump or shade [10]. Fluoroscopic real-time tumor tracking radiotherapy following 4D treatment planning was developed and shown to be feasible to improve the accuracy of the radiotherapy for mobile tumors [11]. In radiation therapy, the projection patterns need to be simple and continuous changes. Because the Fourier transform profile method has significant advantages in noise suppression and full field measurement and the sinusoidal fringe patterns projected by this method meet the requirements of radiotherapy, the Fourier transform profile method is suitable for measuring the 3D motion of the thoracic and abdominal surfaces. However, when only one stripe pattern is projected to measure the entire chest and abdomen surface, the difference in light intensity between adjacent pixels is small due to the large measurement range, and the anti-interference ability is low [12].

In this paper, a dynamic 3D measurement approach of the human chest and abdomen surface during respiration is proposed, which provides a basis for inferring and tracking tumor respiration movement during radiotherapy. This approach adopts a single color stripe pattern with three periods. Through combining one coded pattern with the three RGB primary colors, the sinusoidal stripe pattern with three different periods can be formed. During measurement, the projection pattern does not change, and the deformed stripe image of the chest and abdomen surface is collected in real time. Then, after color coupling correction and color separation, the single color deformation fringe images with three different periods can be formed. The proposed approach can obtain three deformed fringe images by one unchanged pattern. Taking each image sequence of single color fringe as a whole, the 3D Fourier fringe analysis (3D-FFA) method is used to extract the folded phase. This method has higher anti-interference ability. The three-frequency time phase unwrapping method is adopted. The absolute phase is obtained by the folding phase of three monochromatic fringes. This method has a large unwrapping range and strong anti-interference ability. According to the principle of triangulation, the 3D coordinates of the chest and abdomen surface are obtained from the absolute phase [13].

Section 2 introduces the proposed 3D measurement system. Section 3 describes the folding phase extraction method. Section 4 derives a three-frequency time phase unwrapping method. Section 5 shows and analyzes the experimental results, and the conclusions are given in Section 6.

## 2. 3D Measurement System Description

**Figure 1:**
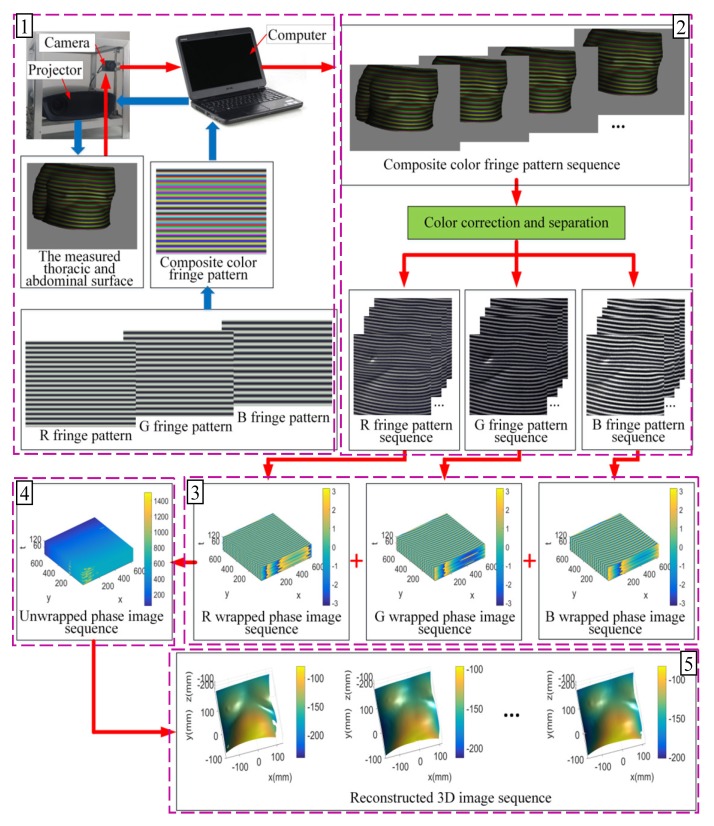
Schematic diagram of the 3D measurement principle and system of the human chest and abdomen surface. (**1**) Pattern projection and image acquisition. (**2**) Image color correction and separation. (**3**) Folded phase extraction. (**4**) Folded phase unwrapping. (**5**) Three-dimensional image sequence acquisition.

Figure 1 is a schematic diagram of the 3D measurement system for the human chest and abdomen surface. The method mainly includes the following five parts.

(1) Pattern projection and image acquisition. The computer generates different periods of three RGB primary color cosine stripe patterns, and these three parts are combined into a composite color stripe pattern, then this pattern is projected to the chest and abdomen surface of the human. The camera captures stripe images of the chest and abdomen surface, which change with breathing movements at regular intervals to get a composite color stripe image sequence. The projection pattern in the proposed approach does not change, which can reduce the time of projection pattern conversion and setup. In addition, the measurement system only collects one composite color stripe pattern, which can decrease the image acquisition time. All these advantages can lay the foundation of the dynamic 3D measurement for the human chest and abdomen surface.
(2) Image color correction and separation. For compound color stripe image sequences, color coupling correction and color separation should be made based on the correction matrix of each pixel, and the three RGB primary color fringe image sequences of different periods can be separated. Because the color coupling phenomenon exists at the coincident intersection of the three color channel spectral response curves in the 3CCD industrial camera [14], the color calibration based on hardware equipment should be completed before the measurement. That is to say, the projector projects four patterns of full red, full green, full blue, and full black to the chest and abdomen surface, and the four images will be captured by the camera. Using these four images, the correction matrix of each pixel is obtained according to the Casti illumination model [15].
(3) Folded phase extraction. For each image in the RGB stripe image sequence, three-dimensional Fourier fringe analysis (3D-FFA) is used to extract the folding phase of each pixel and get the folded phase map of each image, and then, RGB folding phase map sequences can be formed. Fourier fringe analysis (FFA) is extended from one-dimensional Fourier fringe analysis (1D-FFA) to two-dimensional Fourier fringe analysis (2D-FFA) by using the properties of 2D fringe images. After this process, useful signals and interference can be separated better. This method becomes an effective measurement for 3D measurement of flat surfaces [16]. In this paper, the image sequences of the chest and abdomen surface are taken as a 3D one, which is analyzed by 3D Fourier transformation. Useful signals and interference can be separated further by increasing the time dimension, so as to reduce the influence of interference and improve the accuracy of measurement.
(4) Folded phase unwrapping. According to the RGB folding phase diagram at the same time, with the proposed method of three-frequency time phase unwrapping in this paper, the folded phase is expanded into a continuous absolute phase, and the absolute phase diagram at that moment is obtained; thus, an absolute phase sequence can be formed. In the phase unwrapping method of this paper, the unwrapping operation depends on the difference of the decimal part of the measured folded phase, which can ensure that the absolute phase error does not exceed the folded phase error under certain conditions. In addition, we can judge whether there is any big error based on the absolute phase value, which can eliminate or reduce the effect of large absolute phase error by eliminating or interpolating operation. The phase unwrapping is achieved by solving the remainder equation set in the maximum range.
(5) Three-dimensional image sequence acquisition. According to the absolute phase diagram sequence, the 3D coordinates are calculated to form a 3D image sequence of the human chest and abdomen surface based on the triangulation principle. The sequence expresses the 3D shape of the human chest and abdomen surface at each sampling moment during respiratory movement.

## 3. Folded Phase Extraction Method

In this paper, 3D-FFA combines 3D Fourier transform with 3D Gauss filtering in the frequency domain to achieve folded phase extraction. 

### 3.1. Folded Phase Extraction Principle

Taking an image in the *R* fringe image sequence as an example, the principle of folded phase extraction is as follows. Firstly, the intensity of fringe image sequences *i_r_*(*x*,*y*,*t*) at different times *t* can be described as:

(1) $$i_{r}(x,y,t)=a_{r}(x,y,t)+b_{r}(x,y,t)\cos[2\pi (f_{x0}x+f_{y0}y+f_{t0}t)+\varphi_{r}(x,y,t)]$$

where x represents the row coordinate of stripe images, y denotes the column coordinate, $a_{r}(x,y,t)$ is the background light intensity, $b_{r}(x,y,t)$ is the modulation of fringes, $f_{x0}$, $f_{y0}$, and $f_{t0}$ are the carrier frequencies in the direction of x, y, and t, and $\varphi_{r}(x,y,t)$ is the phase distribution function. Equation (1) can be further expressed as:

(2) $$\begin{array}{l} i_{r}(x,y,t)=a_{r}(x,y,t)+d_{r}(x,y,t)\exp[j2\pi (f_{x0}x+f_{y0}y+f_{t0}t)]+\\ d_{r}{}^{*}(x,y,t)\exp[-j2\pi (f_{x0}x+f_{y0}y+f_{t0}t)] \end{array}$$

where:

(3) $$d_{r}(x,y,t)=\frac{1}{2}b_{r}(x,y,t)\exp[j\varphi_{r}(x,y,t)]$$

(4) $$d_{r}{}^{*}(x,y,t)=\frac{1}{2}b_{r}(x,y,t)\exp[-j\varphi_{r}(x,y,t)]$$

After 3D Fourier transform of Equation (2), we can obtain:

(5) $$I_{r}(f_{x},\;f_{y},\;f_{t})=A_{r}(f_{x},\;f_{y},\;f_{t})+D_{r}(f_{x}-f_{x0},\;f_{y}-f_{y0},\;f_{t}-f_{t0})+D_{r}{}^{*}(f_{x}+f_{x0},\;f_{y}+f_{y0},\;f_{t}+f_{t0})$$

where $f_{x0}$, $f_{y0}$, and $f_{t0}$ are the frequency domain variables in the direction of axes x, y, and t, respectively, $A_{r}\left(f_{x},f_{y},f_{t}\right)$ is the background light spectrum, and $D_{r}(f_{x}-f_{x0},\;f_{y}-f_{y0},\;f_{t}-f_{t0})$ and $D_{r}{}^{*}(f_{x}+f_{x0},\;f_{y}+f_{y0},\;f_{t}+f_{t0})$ are the spectra of deformed fringes.

In addition, a 3D filter is used to separate the first level spectrum of $D_{r}(f_{x}-f_{x0},\;f_{y}-f_{y0},\;f_{t}-f_{t0})$ and move it to the origin of the frequency domain. After obtaining $D_{r}(f_{x},\;f_{y},\;f_{t})$, the 3D inverse Fourier transform is performed, and the phase distribution function is as follows,

(6) φr(x,y,t)=tan−1Im{dr(x,y,t)}Re{dr(x,y,t)}

where Im{dr(x,y,t)} denotes the imaginary part of $d_{r}(x,y,t)$ and Re{dr(x,y,t)} is the real part. Similarly, $\varphi_{g}(x,y,t)$ and $\varphi_{b}(x,y,t)$ can be obtained.

### 3.2. Three-Dimensional Gauss Filter

**Figure 2:**
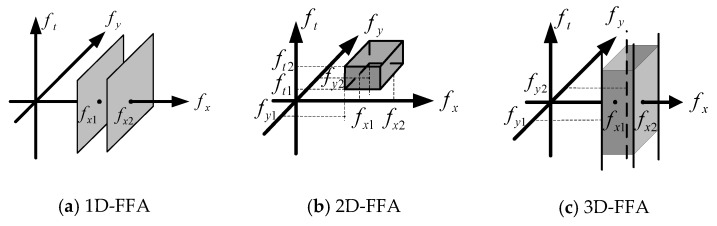
Schematic diagram of filtering in the frequency domain with three methods. FFA, Fourier fringe analysis.

When extracting the positive first-order spectrum of fringe image sequences, the 3D filter must also have the function of filtering interference, which is important for 3D Fourier analysis. Because of the interference existing in the environment, the measured object and the measurement system, and the spectrum leakage led by signal truncation, the folding phase error will happen, which will cause 3D measurement errors. Currently, 3D-FFA mainly uses two types of 3D filters [17]: one is the 3D rectangular filter, and the other is the 3D Butterworth filter [18,19]. The former has truncation problems and large leakage errors. For phase unwrapping, the latter has many problems such as a complex algorithm, accumulated error, and unreliability. By comparison, the Gauss filter has the advantages of a small ringing effect and a good effect of eliminating spectrum leakage. For the determined chest and abdomen surface of the measured human, the center frequency and the width of filter in the 3D direction are determined by experiments, which can make it have good adaptability and filter performance. Moreover, previous studies proved that the effect of the 2D Gauss filter is better than the Hanning window and rectangular window [20]. In this paper, the 3D Gauss filter is used as follows,

(7) $$H(f_{x},f_{y},f_{t})=e^{-\left[\frac{{\left(f_{x}-f_{0x}\right)}^{2}}{2\sigma_{x}^{2}}+\frac{{\left(f_{y}-f_{0y}\right)}^{2}}{2\sigma_{y}^{2}}+\frac{{\left(f_{t}-f_{0t}\right)}^{2}}{2\sigma_{t}^{2}}\right]}$$

where $f_{x0}$, $f_{y0}$, and $f_{t0}$ denote the center frequency of the direction of the x, y, and t axis, respectively, and $\sigma_{x}$, $\sigma_{y}$, and $\sigma_{t}$ represent the filter widths in the three directions, respectively. Figure 2 gives the schematic diagram of filtering in the frequency domain with 1D-FFA, 2D-FFA, and 3D-FFA. As shown in Figure 2, the cut-off frequency in the *x*-axis, *y*-axis, and *z*-axis directions are $f_{x1}=f_{x0}-\sigma_{x}/2$ and $f_{x2}=f_{x0}+\sigma_{x}/2$, $f_{y1}=f_{y0}-\sigma_{y}/2$ and $f_{y2}=f_{y0}+\sigma_{y}/2$, and $f_{t1}=f_{t0}-\sigma_{t}/2$ and $f_{t2}=f_{t0}+\sigma_{t}/2$, respectively. The cut-off frequency value is determined experimentally for each measured object, so that the measured signal passes through as much as possible, and the interference signal passes as little as possible.

In principle, 3D-FFA filters have stronger anti-interference ability than 1D-FFA and 2D-FFA. The 3D shock interference is taken as an example to explain this theory. The spectrum amplitude of the shock interference δ(x,y,t) obtained by 3D Fourier transform is one, and its frequency components cover the entire 3D frequency domain. 1D-FFA can only be filtered along the $f_{x}$ axis in the one-dimensional frequency domain, and its pass band is $f_{x1}<f_{x}<f_{x2}$. As shown in Figure 2a, it can only filter out interference signals in the one-dimensional frequency domain. 2D-FFA filters along the $f_{x}$ axis and $f_{y}$ axis in the 2D frequency domain, the pass bands are $f_{x1}<f_{x}<f_{x2}$ and $f_{y1}<f_{y}<f_{y2}$. As shown in Figure 2b, it can filter out interference signals in the 2D frequency domain, which can further significantly weaken the interference signal. 3D-FFA filters along the $f_{x}$, $f_{y}$, and $f_{t}$ axis in the 3D frequency domain, and the pass bands are $f_{x1}<f_{x}<f_{x2}$, $f_{y1}<f_{y}<f_{y2}$, and $f_{t1}<f_{t}<f_{t2}$, respectively. As shown in Figure 2c, it can filter out interference signals in the 3D frequency domain, which can weaken the interference signal once again.

Due to adding the relationship between adjacent fringe images in time sequence, we can get the Gaussian filter parameters with individual characteristics by designing and optimizing the filter parameters to reduce the ringing effect and spectrum leakage; this can improve the accuracy of wrapped phase extraction. Moreover, 3D-FFA processes all the images at the same time, so it has high efficiency and is suitable for dynamic measurement.

## 4. Tri-Frequency Time Phase Unwrapping Method

Firstly, positive integers $P_{r}$, $P_{g}$, and $P_{b}$ ($P_{r}<P_{g}<P_{b}$) are used to represent the fringe period of the R, G, and B stripe patterns; then $r_{r}$, $r_{g}$, and $r_{b}$ are used to denote the folding phases of the R, G, and B stripe images, and they are the solutions of $\varphi_{r}$, $\varphi_{g}$, and $\varphi_{b}$, respectively in the main value intervals, whose value range is $\left[0,P_{r}\right]$, $\left[0,P_{g}\right]$, and $\left[0,P_{b}\right]$. Therefore, there are the following equations.

(8) $$\varphi_{r}\times P_{r}/(2\times \pi )=r_{r}(\mathrm{mod}P_{r})$$

(9) $$\varphi_{g}\times P_{g}/(2\times \pi )=r_{g}(\mathrm{mod}P_{g})$$

(10) $$\varphi_{b}\times P_{b}/(2\times \pi )=r_{b}(\mathrm{mod}P_{b})$$

In these equations, $P_{r}$, $P_{g}$, and $P_{b}$ are the three modulus values, and $r_{r}$, $r_{g}$, and $r_{b}$ are the corresponding three remainders. 

In addition, N represents the distance between the measured pixel point and the phase origin, which also denotes the absolute phase after the unwrapping processing. Let $N_{r}=[N/P_{r}]$, $N_{g}=[N/P_{g}]$, $N_{b}=[N/P_{b}]$, where [ ] represents a rounding down operation, then *N* can be written as follows:

(11) $$N=N_{r}P_{r}+r_{r}=N_{g}P_{g}+r_{g}=N_{b}P_{b}+r_{b}$$

If $P_{r}$, $P_{g}$, and $P_{b}$ have the greatest common divisor P, there are $\Omega_{r}=P_{r}/P$, $\Omega_{g}=P_{g}/P$, $\Omega_{b}=P_{b}/P$, $\Omega =\Omega_{r}\times \Omega_{g}\times \Omega_{b}$, $\lambda_{r}=\Omega /\Omega_{r}$, $\lambda_{g}=\Omega /\Omega_{g}$, and $\lambda_{b}=\Omega /\Omega_{b}$. Assuming that $\Omega_{r}$, $\Omega_{g}$, and $\Omega_{b}$ are mutually prime, then $\lambda_{r}$ and $\Omega_{r}$ are relatively prime, that is the modulus inverse ${\bar{\lambda }}_{r}$ of $\lambda_{r}$ exists for $\Omega_{r}$, and there is ${\bar{\lambda }}_{r}\lambda_{r}\equiv 1(\mathrm{mod}\Omega_{r})$. For the same reason, there are ${\bar{\lambda }}_{g}\lambda_{g}\equiv 1(\mathrm{mod}\Omega_{g})$ and ${\bar{\lambda }}_{b}\lambda_{b}\equiv 1(\mathrm{mod}\Omega_{b})$.

Let:

(12) $$q_{r}=\left[r_{r}/P\right],q_{g}=\left[r_{g}/P\right],q_{b}=\left[r_{b}/P\right]$$

Then, there is:

(13) $$r_{r}=q_{r}P+r^{c},r_{g}=q_{g}P+r^{c},r_{b}=q_{b}P+r^{c}$$

where $N\equiv r^{c}(\mathrm{mod}P)$ and $N_{0}=[N/P]$, then:

(14) $${\bar{\lambda }}_{r}\lambda_{r}q_{r}+{\bar{\lambda }}_{g}\lambda_{g}q_{g}+{\bar{\lambda }}_{b}\lambda_{b}q_{b}=N_{0}(\mathrm{mod}\Omega )$$

so:

(15) $$N=PN_{0}+r^{c}$$

It is obvious that the folding phases of the R, G, and B fringe images, i.e., the remainders $r_{r}$, $r_{g}$, and $r_{b}$, can be used to unwrap the phase and obtain the absolute phase by Equations (12)–(15).

However, the above discussion does not consider the effect of the residual measurement errors. According to Equation (14), if there are measurement errors $\Delta r_{r}$, $\Delta r_{g}$, and $\Delta r_{b}$ in the folding phases $r_{r}$, $r_{g}$, and $r_{b}$, the errors $\Delta q_{r}$, $\Delta q_{g}$, and $\Delta q_{b}$ belonging to $q_{r}$, $q_{g}$, and $q_{b}$, respectively will be produced, i.e., $\left|\Delta q_{r}\right|\geq 1$, $\left|\Delta q_{g}\right|\geq 1$, and $\left|\Delta q_{b}\right|\geq 1$. In the meantime, $\left({\bar{\lambda }}_{r}\lambda_{r}q_{r}+{\bar{\lambda }}_{g}\lambda_{g}q_{g}+{\bar{\lambda }}_{b}\lambda_{b}q_{b}\right)$ will also produce coarse errors, i.e., $\left|\Delta q_{r}{\bar{\lambda }}_{r}\lambda_{r}\right|\geq {\bar{\lambda }}_{r}\times \Omega_{g}\times \Omega_{b}$, $\left|\Delta q_{g}{\bar{\lambda }}_{g}\lambda_{g}\right|\geq {\bar{\lambda }}_{g}\times \Omega_{r}\times \Omega_{b}$, and $\left|\Delta q_{b}{\bar{\lambda }}_{b}\lambda_{b}\right|\geq {\bar{\lambda }}_{b}\times \Omega_{r}\times \Omega_{g}$. All these can lead the $N_{0}$ and N to produce coarse errors $\Delta N_{MAX}\approx \Delta N_{0MAX}$. Under the condition of ${\bar{\lambda }}_{r}\geq 3$, ${\bar{\lambda }}_{g}\geq 3$, and ${\bar{\lambda }}_{b}\geq 3$, there is $\left|\Delta N_{MAX}\right|\geq 3\times \Omega_{r}\times \Omega_{g}$. Consider that the pixel resolution of the existing device is usually limited within $\Omega_{b}\leq 9$, i.e., $\left|\Delta N_{MAX}\right|\geq \Omega /3$│. $\Delta N_{MAX}$ may further cause $\left({\bar{\lambda }}_{r}\lambda_{r}q_{r}+{\bar{\lambda }}_{g}\lambda_{g}q_{g}+{\bar{\lambda }}_{b}\lambda_{b}q_{b}\right)$ to make errors in the remainder operation with the modulo $\Omega =\Omega_{r}\times \Omega_{g}\times \Omega_{b}$, which may result in a larger absolute phase error. To sum up, $\Delta N_{MAX}$ can cause a large error in the measurement or even lead the measurement to fail. Therefore, some measures should be adopted to avoid the appearance of $\Delta N_{MAX}$ or reduce its influence.

To avoid the appearance of $\Delta N_{MAX}$, assume that the remainder measurement error satisfies Condition A,

(16) $$\left|\Delta r_{r}/P\right|<0.25,\left|\Delta r_{g}/P\right|<0.25,\left|\Delta r_{b}/P\right|<0.25$$

The measured values ${\hat{r}}_{r}$, ${\hat{r}}_{g}$, and ${\hat{r}}_{b}$ of the remainders $r_{r}$, $r_{g}$, and $r_{b}$ can be expressed as:

(17) $${\hat{r}}_{r}/P=\left[{\hat{r}}_{r}/P\right]+\left\{{\hat{r}}_{r}/P\right\},{\hat{r}}_{g}/P=\left[{\hat{r}}_{g}/P\right]+\left\{{\hat{r}}_{g}/P\right\},{\hat{r}}_{b}/P=\left[{\hat{r}}_{b}/P\right]+\left\{{\hat{r}}_{b}/P\right\}$$

where { } is a fractional operation. Then, if the difference between the fractional part of the remainder measurement satisfies Condition B, then:

(18) $$\begin{array}{l} \left|\Delta r_{rg}/P\right|=\left|\left\{{\hat{r}}_{r}/P\right\}-\left\{{\hat{r}}_{g}/P\right\}\right|<0.5\\ \left|\Delta r_{rb}/P\right|=\left|\left\{{\hat{r}}_{r}/P\right\}-\left\{{\hat{r}}_{b}/P\right\}\right|<0.5\\ \left|\Delta r_{gb}/P\right|=\left|\left\{{\hat{r}}_{g}/P\right\}-\left\{{\hat{r}}_{b}/P\right\}\right|<0.5 \end{array}$$

Then, according to the following equation, $q_{r}$, $q_{g}$, and $q_{b}$ can be obtained as follows:

(19) $$q_{r}=\left[{\hat{r}}_{r}/P\right],q_{g}=\left[{\hat{r}}_{g}/P\right],q_{b}=\left[{\hat{r}}_{b}/P\right]$$

Otherwise, according to the following formula, $q_{r}$, $q_{g}$, and $q_{b}$ can be calculated:

(20) $$q_{r}=\left[{\hat{r}}_{r}/P+0.5\right],q_{g}=\left[{\hat{r}}_{g}/P+0.5\right],q_{b}=\left[{\hat{r}}_{b}/P+0.5\right]$$

This ensures that $\Delta q_{r}=0$, $\Delta q_{g}=0$, $\Delta q_{b}=0$, then $\Delta N_{0}=0$, which can eliminate the coarse error $\Delta N_{MAX}$.

According to Equation (14), $N_{0}$ is obtained from $q_{r}$, $q_{g}$, and $q_{b}$, then the absolute phase can be obtained by the following equation:

(21) $$N=PN_{0}+\frac{\left\{{\hat{r}}_{r}/P\right\}+\left\{{\hat{r}}_{g}/P\right\}+\left\{{\hat{r}}_{b}/P\right\}}{3}P$$

According to the above equation, the absolute phase error $\Delta N=\frac{\Delta r_{r}+\Delta r_{g}+\Delta r_{b}}{3}$ does not exceed the folding phase errors $\Delta r_{r}$, $\Delta r_{g}$, and $\Delta r_{b}$.

If the residual measurement error does not satisfy Condition A, $\Delta N_{MAX}$ may occur. In order to reduce the influence of $\Delta_{MAX}$, the proposed method in this paper increases the part of judging and processing the absolute phase. After the phase unwrapping is completed, the difference $\Delta N_{k}=N_{k}-N_{k-1}$ between the absolute phase measurement value $N_{k}$ of each pixel k and the absolute phase measurement value $N_{k-1}$ of its neighboring pixel is calculated. If it meets the following Condition C,

(22) $$\left|\Delta N_{k}\right|<3\times \Omega_{r}\times \Omega_{g}$$

We regarded $N_{i}$ as the valid measurement value, and the absolute phase error does not exceed the folding phase errors $\Delta r_{r}$, $\Delta r_{g}$, and $\Delta r_{b}$; otherwise, $\left|\Delta N_{k}\right|\geq 3\times \Omega_{r}\times \Omega_{g}$│, which means the spatial distance between adjacent pixels is no less than 1/3 of the range. This is obviously unreasonable for a relatively flat surface such as the chest and abdomen surface of the human body. If $N_{i}$ is invalid, the pixel can be rejected as an immeasurable point.

If necessary, the interpolation method can be used to obtain the absolute phase based on the surrounding pixels of the absolute phase. Elimination or interpolation usually has little effect on the measurement because the sample points in the image are large and dense. Conversely, if a larger absolute phase error cannot be identified and eliminated, it will affect the measurement result seriously or even make the measurement result unusable. This is also a challenging problem in the 3D measurement of Fourier fringe analysis [21]. To sum up, under Conditions of A, B, and C, Equations (19)–(21) are combined to form our proposed 3D measurement method.

In addition, compared with the three-frequency differential method, the proposed method has a larger unwrapping range. Let $P_{r}=P_{0}-W_{r}$, $P_{g}=P_{0}$, $P_{b}=P_{0}+W_{b}$, where $W_{r}$ and $W_{b}$ are positive integers, and the phase unwrapping range of the proposed method is $P_{rgbO}=P_{r}P_{g}P_{b}$. When phase unwrapping is performed by the three-frequency differential method, the light stripes R and G are used for phase unwrapping to form a synthetic light stripe RG with the phase unwrapping range $P_{rg}=P_{r}P_{g}/(P_{g}-P_{r})$; then, use the light stripes G and B for phase unwrapping to form a synthetic light stripe GB with a phase unwrapping range $P_{gb}=P_{g}P_{b}/(P_{b}-P_{g})$; moreover, the phase unwrapping is further performed by using the synthesized light stripes RG and GB. When $P_{gb}>P_{rg}$, its phase unwrapping range is $P_{rgbH}=P_{r}P_{g}P_{b}/(2W_{r}W_{b}+(W_{r}-W_{b})P_{0})$. When $W_{r}\geq W_{b}$, $2W_{r}W_{b}+(W_{r}-W_{b})P_{0}\geq 2$, then $P_{rgbO}\geq 2P_{rgbH}$. Only when $W_{r}<W_{b}$, $2W_{r}W_{b}+(W_{r}-W_{b})P_{0}=1$, that is $P_{rgbO}=P_{rgbH}$ is possible. According to the pixel resolution of the currently available digital pattern projection device and digital image acquisition device, the phase unwrapping range should be 300 to 10,000 pixels. Only in these two cases, one being $P_{r}=9\;\mathrm{pixel}$, $P_{g}=11\;\mathrm{pixel}$, $P_{b}=14\;\mathrm{pixel}$, $P_{rgbH}=1386\;\mathrm{pixels}$ and the other being $P_{r}=12\;\mathrm{pixel}$, $P_{g}=17\;\mathrm{pixel}$, $P_{b}=29\;\mathrm{pixel}$, $P_{rgbH}=5916\;\mathrm{pixels}$, there will be $P_{rghO}=P_{rgbH}$. Under these situations, it is difficult to achieve comprehensive optimization of the measurement range, resolution, and anti-interference ability by flexible selection of $P_{r}$, $P_{g}$, and $P_{b}$.

## 5. Experimental Results and Analysis

### 5.1. Simulation Experiments of Folding Phase Extraction

Ethical approval to undertake this project was examined by the Human Research Ethics Committee for Non-Clinical Faculties, School of Measurement-Control Technology and Communication Engineering, Harbin University of Science and Technology on 1 March 2019. The title of the project is “Projection on Patient Body Surface in Invasive Surgeries (National Natural Science Foundation of China, 61671190)”. Informed consent form was obtained from the subject. Simulation experiments were conducted by using a planar cosine light image sequence with a size of 768 × 768 pixels and a period $P_{0}$ of 35 pixels. To simulate human respiratory movement that approximates periodic motion, let the measured plane do relative paralleled movement to the image plane of the camera 2mm each time and perform a periodic reciprocating motion with a period of 20mm, then collect the plane cosine light images from 120 positions.

(23) $$i_{m}(x,y,t)=128+75\times \cos[2\pi x/P_{0}+2\pi (t-1)/20]\;m=1,2,3,\cdots ,\;120.$$

**Figure 3:**
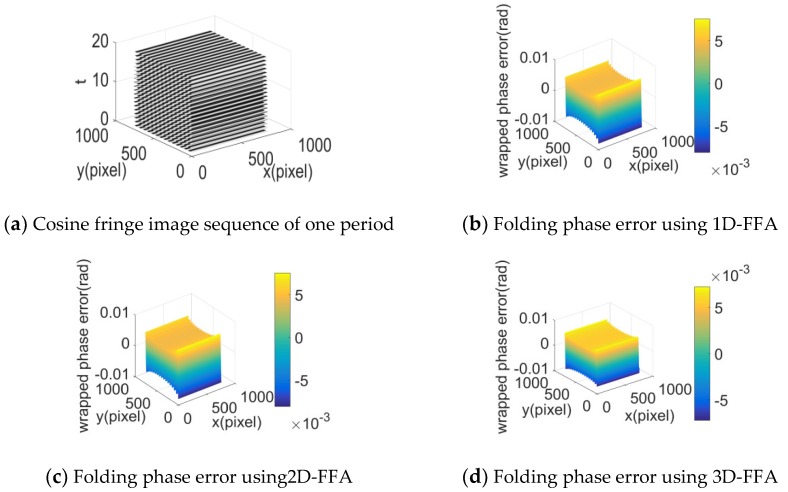
Cosine light image sequence and folding image error of one image.

The 3D representation of a cosine fringe image sequence with one period in the t-axis direction is shown in Figure 3a. Extract the folding phases of the sixth frame image by 1D-FFA, 2D-FFA, and 3D-FFA, respectively. Then, these extracted folded phases are subtracted from the folded phase, respectively. The folding phase errors of the three methods are shown respectively in Figure 3a–c.

According to Figure 3, in the extracted folded phase by the three methods, there were errors caused by truncation and sampling. The calculated folding phase value of measured points and their standard values were used to calculate the RMS error, and the difference between the maximum folding phase value and the minimum folding phase value was defined as peak-to-valley (PV) error. As shown in Table 1, the PV and RMS of the folded phase error were approximately equal, and they were so small that could be neglected. It can be seen that these three methods could extract the folding phase effectively.

To evaluate the anti-interference ability of 3D-FFA, the following interference signals were added to the cosine fringe image sequence:

(24) $$\gamma =75\times I_{p}\times \left[2\times \mathrm{rand}(768,768,120)-1\right]$$

where rand() is a function of generating random numbers in [0,1] and $I_{p}$ is the ratio percentage of the interference signal amplitude to the cosine modulation.

By analyzing the fringe images collected in the experiments, the results showed that the noise mainly consisted of salt and pepper noise and Gaussian noise, whose probability distribution curves were superimposed and integrated to form a uniform noise probability curve. Therefore, the uniformly distributed random noise could be used in the simulation experiment, which could fully simulate the effect of noise and was better than the direct superposition of Gaussian noise and salt and pepper noise. 

**Figure 4:**
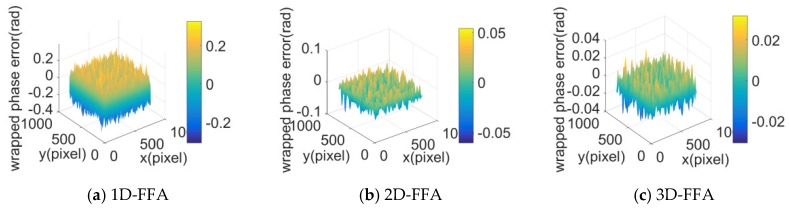
Folding phase error of a stripe image after adding interference.

Taking $I_{p}=60\%$ as an example, the folding phase error of the sixth frame image is shown in Figure 4. It can be seen that the folding phase error extracted by 3D-FFA was obviously smaller than that of the other two methods, which showed that the anti-interference ability of the 3D-FFA was the strongest.

**Figure 5:**
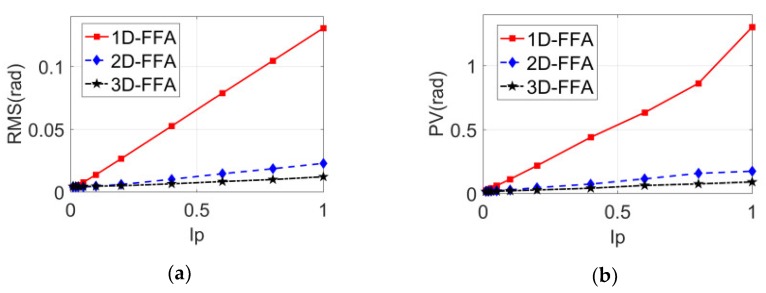
The folded phase error curves of the sixth frame stripe images with different interference percentages. (**a**) Comparison of the root mean square of the folded phase error; (**b**) comparison of peak and valley values of folded phase errors.

When $I_{p}$ was 1%, 2%, 3%, 5%, 10%, 20%, 40%, 60%, 80%, and 100%, respectively, the folded phase error curves of the three methods are shown in Figure 5. As far as the RMS error and peak-valley error of folded phase were concerned, one was that they increased with the increase of interference; the other was that the results obtained by the 2D-FFA method were significantly lower than the 1D-FFA method; the third was that the results obtained by 3D-FFA method were significantly lower than the 2D-FFA method; the fourth was that the 3D-FFA method and the 2D-FFA method were almost invariant and approximate to the interference when the interference ratio was less than 10%.

**Figure 6:**
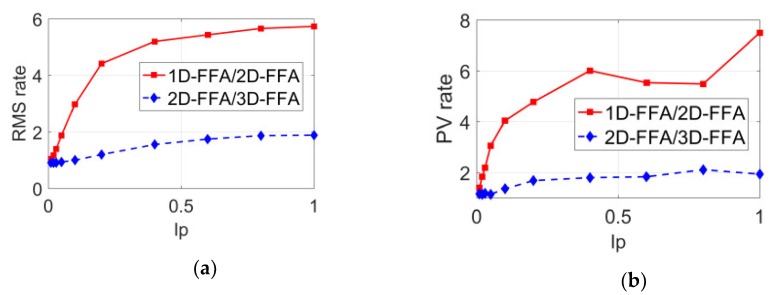
The ratio curves between the folded phase errors of the three methods with different interference ratios of the sixth frame. (**a**) RMS ratio curve of folded phase error; (**b**) PV ratio curve of folded phase error.

In order to compare the anti-interference ability of the three methods quantitatively, the ratio of the folding phase errors of the three methods with different interference percentages is shown in Figure 6. In Figure 6a, the red line is the RMS error ratio of 1D-FFA and 2D-FFA, which was between four and six; the blue line is the RMS error ratio of 2D-FFA and 3D-FFA, which was between one and two. In Figure 6b, the red line is the PV error ratio of 1D-FFA and 2D-FFA, which was between four and eight; the blue line is the PV error ratio of 2D-FFA and 3D-FFA, which was between one and two. Obviously, the anti-interference capability of 2D-FFA method was much better than that of the 1D-FFA method, and the one of the 3D-FFA method was much better than that of the 2D-FFA method; the bigger the interference, the more obvious the superiority of the anti-interference capability. When the interference reached 40%, the anti-interference ability of the 3D-FFA method was about twice that of the 2D-FFA method. 

### 5.2. Chest Model Measurement Experiments

Based on the proposed method in this paper, a 3D experimental apparatus was constructed for human chest and abdomen surface measurement. The device used a projector (InFocus IN82, InFocus Corporation, Wilsonville, OH, USA) to project a color stripe pattern with a resolution of 1024 × 768 pixels. The pattern parameter was $P_{r}=25\;\mathrm{pixel}$, $P_{g}=30\;\mathrm{pixel}$, $P_{b}=35\;\mathrm{pixel}$, and the measured surface stripe images with a resolution of 1624 × 1236 pixels were collected by using a 3CCD industrial camera (AT-200GE, JAI Ltd., Copenhagen, Denmark).

**Figure 7:**
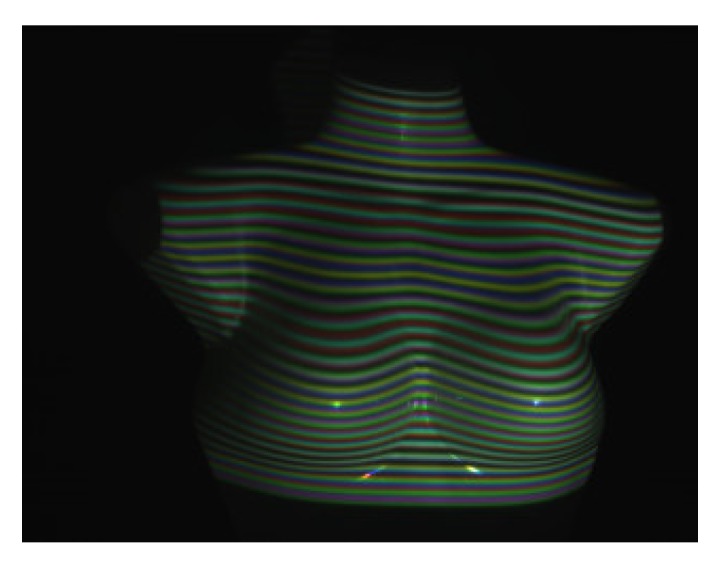
Image of the chest model after the projected stripe pattern.

**Figure 8:**
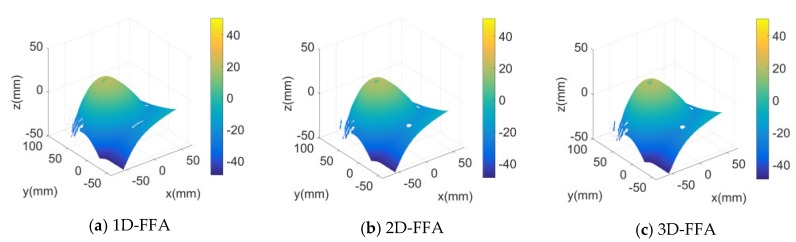
Measurement results of the sixth frame stripe image of the chest model.

In the measurement experiments, the chest model simulated respiratory movement and reciprocating motion on the guide rail for 20 mm. For each mobile 2 mm, we collected an image as shown in Figure 7. A total of 120 images was collected and sent to the computer to form a sequence of stripe images. Intercept the stripe image sequence with the size of 768 × 768 pixels from the fringe image sequence, and then get the 3D image sequence of the tested area by using the 3D measurement method. Figure 8a–c shows the sixth frame of 3D images formed by taking folded phases using 1D-FFA, 2D-FFA, and 3D-FFA, respectively. All of them could reproduce the 3D surface of the measured area correctly, which verified the 3D measurement method presented in this paper. The visual effect of the three methods was basically the same, because the measurement errors of the three were basically the same when the interference could be ignored in the darkroom.

**Figure 9:**
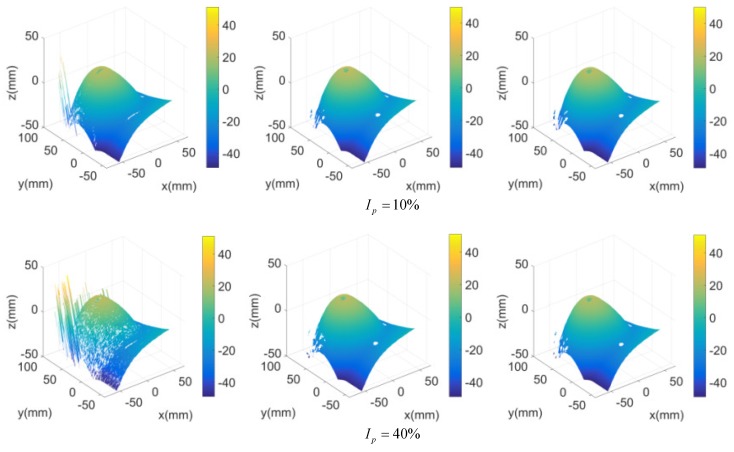

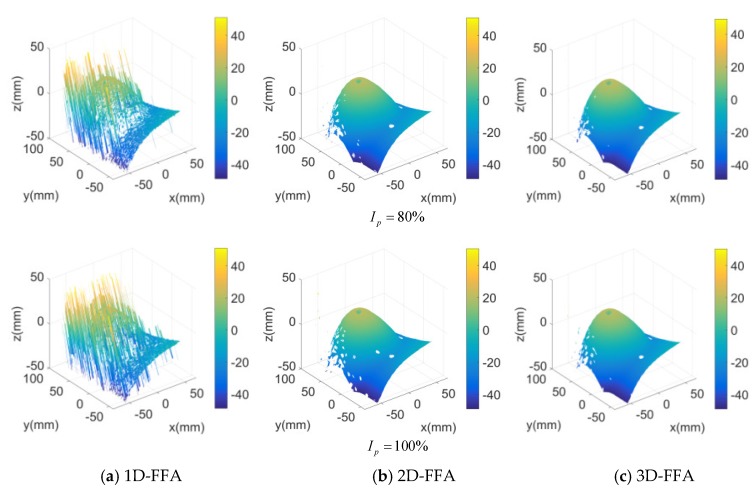
Measurement results of the sixth stripe image of the chest model with different interference.

In order to verify and compare the anti-interference ability of the 3D measurement method in this paper, we added the interference γ of different $I_{p}$ to the stripe sequence of the chest model. Take the sixth frame stripe image as an example, the measurement results are shown in Figure 9. When the interference was 5%, the error of the three measurements was similar. 

Beginning with $I_{p}=10\%$, the error of 1D-FFA measurement result increased rapidly, and the errors of the other two measurements also increased. The error of measurement based on 2D-FFA was larger than 3D-FFA in the range of 10%–80% for $I_{p}$, although it was difficult to observe visually. From the beginning of $I_{p}=80\%$, the error of measurement based on 2D-FFA was larger than that based on 3D-FFA. It showed that the proposed method had the strongest anti-interference ability, and the greater the interference, the more obvious the advantage. 

### 5.3. Measurement Experiments of the Human Chest and Abdomen Surface

**Figure 10:**
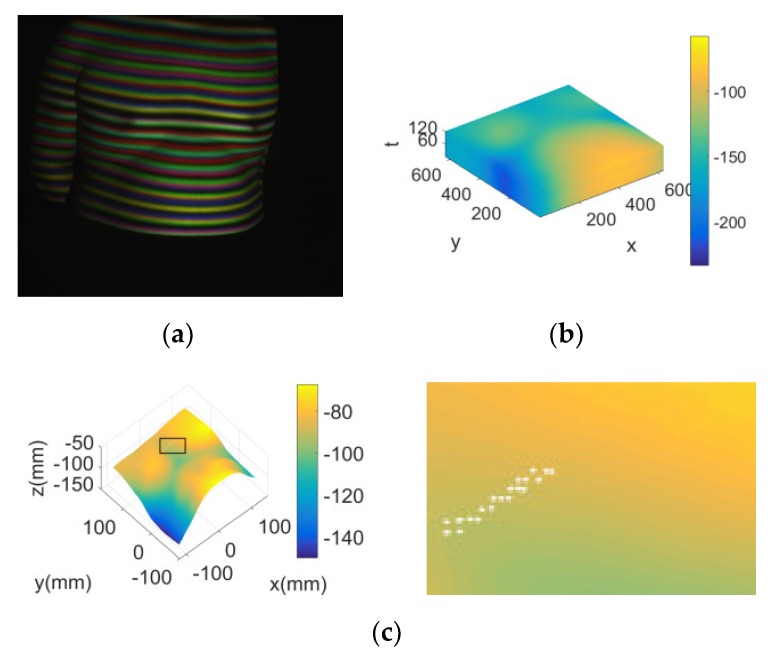

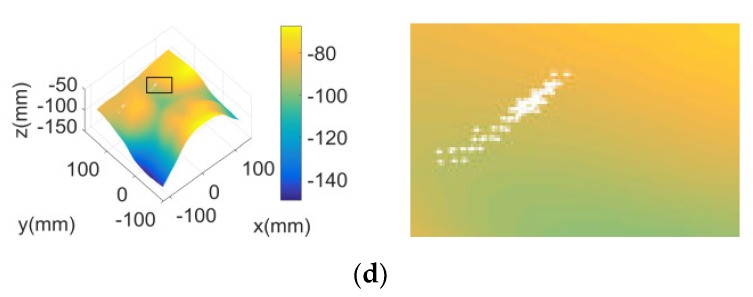
Surface of the human chest and abdomen and its measurement results. (**a**) Measured surface of the chest and abdomen; (**b**) 3D surface depth image sequence of the measured area; (**c**) measurement results of the sixth stripe image and the local magnification map when ignoring the interference; (**d**) measurement results of the sixth stripe image and the local magnification map after adding 30% interference.

With the proposed 3D measurement method, a 3D measurement experiment of the chest and abdomen surface was conducted during its respiratory movement, as shown in Figure 10a, and the 3D surface depth image sequence of the measured area is shown in Figure 10b. The sixth frame in the 3D image sequence is shown in Figure 10c. That is to say that, the proposed method could reconstruct the 3D surface of the human chest and abdomen correctly. In Figure 10c, the enlarged part of the square area on the left is shown on the right, which indicated that there was maximum measurement error in the indoor light environment, which formed an undetectable blank spot.

In the measurement process, interference was added artificially. When the interference was small, the measurement results were basically unchanged. When the interference reached 30%, the measurement results are shown in Figure 10d, which indicated that the undetectable points increased significantly, and the measurement results started to become significantly worse. If necessary, the measured value of the white area could be obtained by the difference between the effective measurement values of its peripheral adjacent points.

**Figure 11:**
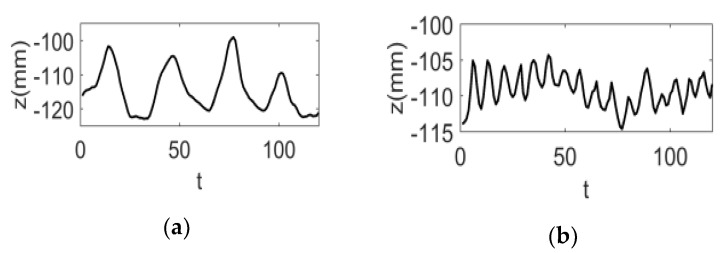
The height (z)-time (t) respiratory motion curve at a point near the diaphragm. (**a**) Curve of the point’s motion in deep breathing; (**b**) curve of the point’s motion in rapid breathing.

However, for a relatively flat surface such as the surface of the human chest and abdomen, eliminating the isolated white spot or obtaining the blank area by the difference had little effect on the measurement results. As shown in Figure 11, a fixed point near the diaphragm was selected, and the height (z) of the point in the image sequence changed with time (t) to form the height (z)-time (t) respiratory movement curve of the point. The measurement results of two typical respiratory movement states, deep breathing and rapid breathing, are given in Figure 11a,b, respectively.

The measured results showed that the proposed method in this paper could recover the 3D shape of the surface of the chest and abdomen at different times correctly, realize the dynamic 3D measurement of the surface, and obtain the respiratory motion trajectory of the surface points. This method had strong anti-interference ability and could eliminate the maximum measurement error or its influence basically.

## 6. Conclusions

In this paper, a 3D dynamic surface measurement method was proposed for the human chest and abdomen. The RGB trichromatic cosine stripe pattern was synthesized into a color stripe projection pattern. The projection pattern was invariable, and one measurement could be realized by collecting one stripe image. It had less pattern projection and image acquisition time, which provided the foundation for dynamic measurement. Color correction and color separation for the color stripe image sequence were used to form the RGB monochromatic deformation stripe image sequence with a different period. The 3D Fourier transform and the 3D Gaussian filter were combined to carry out 3D-FFA to extract the folding phase. The interference effect could be reduced, and the measurement accuracy could be improved by increasing the time dimension frequency domain filter. The phase unwrapping operation depended on the difference between the fractional parts of the folded phase error, which could ensure that the absolute phase error did not exceed the measurement error of the remainder. Removal or interpolation based on absolute phase measurement could eliminate or reduce the effect of gross absolute phase error. The phase unwrapping based on the remainder equation had a larger phase unwrapping range and preferred space.

In view of this proposed method, the theoretical analysis and experimental results showed that the method presented in this paper could obtain the 3D image sequence of the human chest and abdomen surface and the respiratory motion curve of the chest and abdomen surface points in the respiratory movement effectively and accurately. It had the characteristics of strong anti-interference ability and a wide range of development and could eliminate gross absolute phase error. It should be pointed out that although the method in this paper achieved the analysis of five frames of 3D images per second and respiratory motion in about four seconds, its operation time should be reduced from both the perspective of improving the analysis effect and engineering demand. Therefore, we plan to adopt multithread optimization measures to improve the operation speed.

## Figures and Tables

**Table 1:** Folding phase error of the stripe image (unit: rad).

Methods	1D-FFA	2D-FFA	3D-FFA
PV	0.0156	0.0156	0.0145
RMS	0.0040	0.0040	0.0044
